# GPR30 Activation Promotes the Progression of Gastric Cancer and Plays a Significant Role in the Anti-GC Effect of Huaier

**DOI:** 10.1155/2022/2410530

**Published:** 2022-01-19

**Authors:** Xiao-feng Wang, Can Hu, Shao-Wei Mo, Jing-Li Xu, Yan Liu, Han-dong Xu, Li Yuan, Ling Huang, Jian-fa Yu, Xiang-Dong Cheng, Zhi-yuan Xu

**Affiliations:** ^1^Second Clinical Medical College, Zhejiang Chinese Medical University, Hangzhou 310053, China; ^2^Affiliated Cixi Hospital, Wenzhou Medical University (Cixi People's Hospital), Ningbo 315300, China; ^3^The Cancer Hospital of the University of Chinese Academy of Sciences (Zhejiang Cancer Hospital), Institutes of Basic Medicine and Cancer (IBMC), Chinese Academy of Sciences, Hangzhou 310022, China; ^4^Diagnosis and Therapy Center of Upper Gastrointestinal Tumor in Zhejiang Province, Hangzhou 310022, China

## Abstract

Gastric cancer (GC) is one of the most common types of cancer. The n-butanol extract of Huaier (NEH) is the alcohol-soluble part extracted by the systematic solvent method, which is effective against gastric cancer (GC). However, the mechanism of action of NEH remains unclear. In this study, we aim to evaluate the clinical relevance of GPR30 expression in GC patients and the role of the GPR30/PI3K/AKT signalling pathway in the anti-GC effect of NEH. The expression of GPR30 was examined using immunohistochemistry. Cell counting kit 8 (CCK-8) assay, wound healing, and transwell experiments were used to investigate the viability, migration, and invasion of gastric cancer cells. Western blotting was used to detect the expression of GPR30 and its downstream signalling molecules of the PI3K/AKT signalling pathway. Gastric cancer patient-derived xenografts (PDX) mouse model was used to evaluate the antitumor effect of NEH in *vivo*. In addition, the graded doses and the maximum tolerated dose of NEH were administered intraperitoneally into the mice for acute toxicity test. We demonstrate that GPR30 expression in GC tissues was significantly higher than that in corresponding adjacent noncancerous tissues and the expression of GPR30 was correlated with a poor prognosis in GC patients. Moreover, GPR30 expression was involved in the migration and invasion of GC cells in vitro. Additionally, we found that NEH can suppress the growth of GC in patient-derived xenograft tumors in vivo. Furthermore, NEH inhibited the proliferation, migration, and invasion in GC cells in a concentration-dependent manner through inhibiting the GPR30-mediated PI3K/AKT signalling pathway in vitro. Acute toxicity test showed that NEH caused no toxic reaction or death and the maximum tolerated dose of NEH in mice was greater than 1600 mg/kg. Our results demonstrate that the high expression of GPR30 is an independent factor of poor prognosis in patients with GC and NEH could be a new agent for the treatment of gastric cancer.

## 1. Introduction

Gastric cancer (GC) is one of the most common types of cancers [1]. Despite significant progress in the treatment of GC, the prognosis remains poor. Two-thirds of patients with GC are already in advanced stage when they are first diagnosed, and there is no chance of surgery. More than 70% of patients with early GC will have recurrence or metastasis after surgery [2]. Therefore, finding effective potential targeted therapy is the key to the treatment of advanced GC.

GPR30, a new type of estrogen receptor, is different from the classic estrogen receptors (ER*α* and ER*β*) in structure and function. It participates in the rapid activation of intracellular signalling transduction pathways through the G protein *βγ* heterodimer and G*α* subunit, including the mobilization of intracellular calcium storage, the transactivation of human epidermal growth factor receptor (EGFR), and the activation of downstream signalling pathways such as PI3K/AKT signalling pathway [3]. Our previous studies demonstrated that GPR30 was a key factor in the regulation of EMT in GC [4]. In addition, some researchers have found that GPR30 mediates the nontranscriptional effect of estrogen on the activation of PI3K/AKT signalling pathway in endometrial cancer [5].

Huaier, which belongs to the class Hymenomycetes, *Phylum Basidiomycota*, is a beige sandy mushroom that grows on the trunk of trees [6–10]. Its antitumor potency was discovered in recent decades. Huaier has been used for the treatment of gastric cancer [11], liver cancer [8], and breast cancer [12], as well as nonsolid tumors such as leukemia [13]. Previous studies confirmed that the aqueous extract of Huaier could block the cell cycle of GC cells in the G2/M phase by inhibiting cyclin B1 expression and induce the apoptosis of GC cells through a PI3K/AKT signalling pathway [14]. However, some of the active components of Huaier are insoluble in water, while temperature affects the activity and composition of its water extract. In our previous study, we improved the extraction method and adopted the systematic solvent method to extract Huaier into five organic phases: petroleum ether, ethyl acetate, n-butanol, ethanol, and water, and identified the *n*-butanol extract of Huaier (named as NEH) as the most effective components against GC in *vitro*. Additionally, we confirmed that NEH was more effective in inhibiting GC cells compared with the aqueous extract of Huaier and was able to significantly enhance the effect of cisplatin in GC cells by reducing the expression of MRP1 (multidrug resistance-associated protein 1). Moreover, our previous work identified the total flavonoids (accounting for 51.4%) as the main active component of NEH [15]. However, the specific molecular mechanism of the anticancer effect of NEH is not clear.

This study investigated the mechanism of action of NEH for its anticancer activity in human gastric cancer both *in vitro* and *in vivo*. We found that NEH significantly inhibited the proliferation, migration, and invasion of gastric cancer cells through the PI3K/AKT signalling pathway mediated by GPR30.

## 2. Materials and Methods

### 2.1. Tissue Microarray (TMA) Construction and Immunohistochemistry (IHC) Analysis

Ninety-one formalin-fixed, paraffin-embedded (FFPE) GC tissues and seventy-one corresponding adjacent noncancerous tissues were collected from the Department of Gastrointestinal Surgery, The First Affiliated Hospital of Zhejiang Chinese Medical University. Two pathologists screened all FFPE GC tissues independently to confirm the diagnosis of GC. The most representative tumor and noncancerous tissues were selected to construct the TMA. The IHC staining was performed according to the previously described procedures [16]. The expression of GPR30 was assessed using the H-score system:

(1) $$\begin{array}{c} H-\text{score}=\sum \left(\text{IS}\times \text{AP}\right), \end{array}$$

where IS represents the staining intensity and AP represents the percentage of positively stained tumor cells, producing a score ranging between 0 and 12. An IS ranging between 0 and 3 was assigned for the intensity of tumor cell staining (0, no staining; 1, weak staining; 2, intermediate staining; and 3, strong staining). AP depended on the percentage of positive-stained cells as follows: 0 (0%), 1 (1–25%), 2 (26–50%), 3 (51–75%), and 4 (75–100%). The score was assigned using the estimated proportion of positively stained tumor cells. Multiple regions were analyzed to assess the average staining score within a tumor sample, and at least 100 tumor cells were assessed. Two researchers who were blinded to the clinical outcomes performed the scoring independently.

### 2.2. Cell Culture and Chemicals

GC cell lines MGC-803, BGC-823, HGC-27, AGS, MKN-45, and normal gastric epithelial cell GES-1 were obtained from the Cell Resource Center, Peking Union Medical College. All cells were cultured in RPMI-1640 medium containing 10% fetal bovine serum (FBS, Hyclone, Utah, USA) in a humidified atmosphere containing 5%CO_2_ at 37°C. G1 (Cat# HY-107216), G15 (Cat# HY-103449), and cisplatin (CDDP, Cat# HY-17394) were obtained from MedChemExpress (New Jersey, USA).

The primary antibodies against GPR30 (Cat# ab260033), MMP2 (Cat# ab92536), and MMP9 (Cat# ab76003) were purchased from Abcam (Cambridge, UK). Anti-PI3K (Cat# 4255S), Anti-phospho-AKT (Ser473, Cat# 4060T), Anti-AKT (Cat# 4691T), Anti-CDK2 (Cat# 18048T), Anti-Cyclin A2 (Cat# 4656T), Anti-Cyclin D1 (Cat# 55506T), Anti-N-cadherin (Cat# 13116T), and Anti-Vimentin (Cat# 5741T) antibodies were purchased from Cell Signal Technology (Boston, USA). Anti-GAPDH (Cat# AF1186) and *β*-actin (Cat# AF5001) antibody were purchased from Beyotime (Shanghai, China).

### 2.3. Cell Proliferation Assay

The cell viability was measured by CCK-8 assay (Dojindo, Japan) following the manufacturer's instructions. Briefly, cells were seeded into 96-well plates at a density of 3 × 10^3^ cells/well before treatment. After 12 h, cells were exposed to the drug for 24, 48, and 96 h, to examine the growth inhibitory effects; 6 replicates were applied at each concentration. At each time point, 10 *μ*l of sterile CCK-8 reagent was added to each well and incubated for 2 h at 37°C. The light absorbance at 450 nm was determined using a microplate reader (Bio-Rad, USA). IC50 values were calculated from the linear regression of the plot.

### 2.4. Western Blot

Total cell proteins were prepared from RIPA Lysis Buffer (Conway Century, Beijing, China) with proteinase inhibitor cocktails (Merck Millipore, USA). The protein concentration was determined by the BCA protein kit (Conway Century, Beijing, China). Subsequently, the same amounts of total proteins (30 *μ*g) were electrophoretically separated using SDS-PAGE and transferred onto PDVF membranes. After blocking, the membranes were incubated with the primary antibodies. After being washed twice by TBST, samples were incubated with anti-rabbit or anti-mouse HRP-conjugated secondary antibodies (Beyotime, Shanghai, China). ECL chemiluminescence method was used to visualize the target proteins.

### 2.5. Wound Healing Assay

The cells were inoculated into Culture-Inserts (Ibidi, Martin Reid, Germany) in 24-well plates at a density of 2 × 10^4^ cells/well. After the cells filled the entire area, the culture-insert was removed. The cells were then rinsed twice with PBS to remove floating cells and incubated in RPMI-1640 medium containing the drug and 1% FBS. Images were taken using an inverted microscope at 0 and 24 h of incubation.

### 2.6. Cell Invasion Assay

The invasive ability of cells was detected by transwell assays. In short, an equal amount of 40 *μ*L of Matrigel was added in the upper chambers of the transwell chambers (8 *μ*m pore size; Corning, Shanghai, China) and placed at room temperature. Then, 6 × 10^4^ cells (200 *μ*l) in a serum-free medium containing drugs were placed in the upper chambers. The RPMI-1640 medium containing 10% FBS was added to the lower chambers. After being incubated for 48 h at 37°C, the cells were fixed with methanol and stained with 0.1% crystal violet. The noninvasive cells in the upper chambers were wiped off with a cotton swab, and the number of invading cells at the bottom of the transmembrane pore was counted using a microscope.

### 2.7. Plant Material and Preparation of Extract

**Figure 1:**
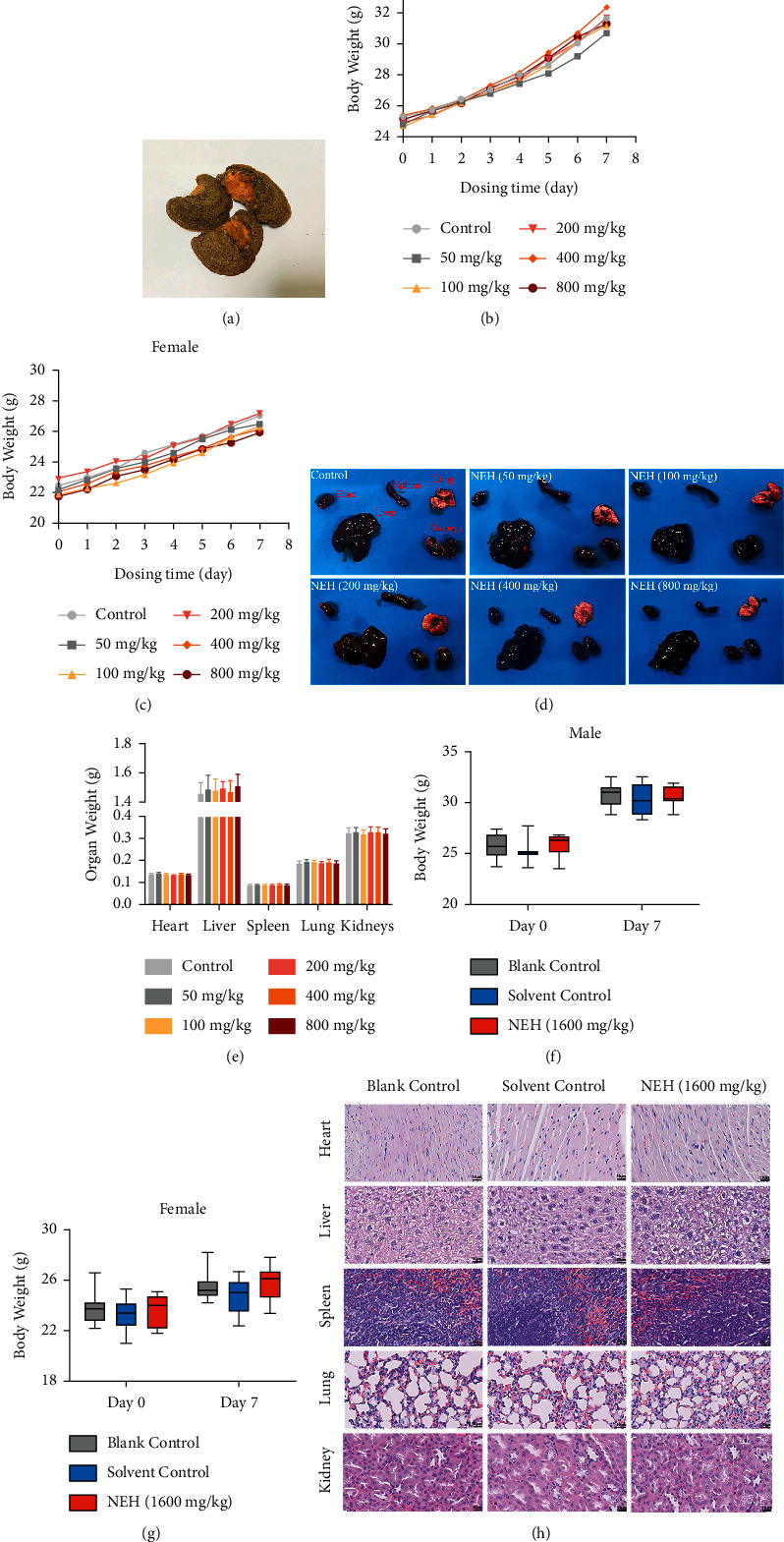
The acute toxicity of NEH in mice. (a) The fruiting bodies of Huaier. (b, c) No significant change in body weight of male (b) and female mice (c) was observed after intraperitoneal injection of 50, 100, 200, 400, and 800 mg/kg/day NEH. *n* = 4 per group. (d-e) The difference of organ weight of heart, liver, spleen, lung, and kidneys in each group. (f, g) There was no significant change in body weight of male (f) and female mice (g) in MTD test. *n* = 20 per group. (h) H&E staining for pathological examination in order to observe the pathological changes of organs in three groups.

As shown in Figure 1(a), the fruiting bodies of Huaier were purchased from the traditional Chinese Medicine Store of Zhejiang Academy of Traditional Chinese Medicine. Huaier was mixed with 90% ethanol, heated, and reflux-extracted twice for 2 h each time. After the extract was concentrated and dried, the ethanol extract was obtained. Then, the ethanol extract was further extracted with petroleum ether, ethyl acetate, *n*-butanol, ethanol, and distilled water, as previously described [17]. Five kinds of powders of Huaier with different polarity were obtained by concentrating, freezing, and vacuum-drying on a rotary evaporator. After being dissolved in DMSO (dimethyl sulfoxide) and phacoemulsified, 400 mg/ml (maximum dissolved dose) stock solution of n-butanol extract of Huaier was obtained and stored at −20°C. After configuration of the diluent, the solution was sterilized with a 0.22 *μ*m filter.

### 2.8. Animals

SPF grade ICR mice and BALB/c nude mice were provided by the Animal Experimental Research Center of Zhejiang University of Traditional Chinese Medicine. All the animals were housed in an environment with a relative humidity of 50 ± 1%, a temperature of 22 ± 1°C, and a light/dark cycle of 12/12 hr. The animals were fasted for 12 h before the start of experiments. All animal studies (including the mouse euthanasia procedure) were performed in compliance with the regulations and guidelines of Zhejiang University of Traditional Chinese Medicine institutional animal care and conducted according to the AAALAC and the IACUC guidelines (Reg No: 20181217–02).

### 2.9. Patient-Derived Xenograft (PDX) Mouse Model

The PDX mouse model was established in BALB/c nude mice using fresh GC tissue removed from the patient (an elderly female patient with poorly differentiated adenocarcinoma with a staging of T4N3M0 who signed the informed consent). The third-generation xenograft tumor was used in the experiment. The tumor-bearing mice were randomly divided into three groups: control group (5% DMSO+95% saline), NEH treatment group (25 mg/kg), and cisplatin treatment group (5 mg/kg). When the tumor volumes reached 100 mm^3^, mice were intraperitoneally injected with saline or drugs 3 times a week for a total of 4 weeks. The body weights and tumor volumes were measured twice a week. At the end of the experiment, all mice were sacrificed for histological examination.

### 2.10. Acute Toxicity Test

An acute toxicity test was conducted using the acute toxicity classification method according to the OECD- (Organization of Economic Corporation and Development-) 423 Guidelines [18]. Twenty-four ICR mice were randomly divided into six groups (2 male and 2 female mice in each group): five groups of mice were intraperitoneally given 50, 100, 200, 400, and 800 mg/kg/day of NEH (10% DMSO+90% saline). The mice of the control group was intraperitoneally injected with equal volume of normal saline (containing 10% DMSO). After administration, the general behavior and toxic signs of mice were continuously observed for 30 min and then intermittently for 4 h, over a period of 24 h. Then, these animals were observed twice a day for 7 days to detect the changes in their body weight, behavior, and toxicity/death. At the end of the 7th day, mortality was calculated as LD_50_ according to Karber's method. When the LD_50_ value could not be determined, mice were intraperitoneally injected with the maximum tolerable dose of NEH for maximum tolerance (MTD) test.

Sixty ICR mice were randomly divided into 3 groups (10 male and 10 female mice in each group): blank control group (saline), solvent control group (10% DMSO+90% saline), and NEH group (1600 mg/kg). The observation method was the same as above.

Blood biochemical analysis was performed after observation. The organs and tissues were fixed, embedded in paraffin, and stained with hematoxylin and eosin (H&E) for histological analysis.

### 2.11. Statistical Analysis

Statistical analysis was performed by using GraphPad Prism 8.0 software. All data were presented as mean ± SEM. Students' 2-tailed *t*-test was used for comparison between the two groups, and ANOVA analysis was used in multiple comparisons. *P* < 0.05 was considered statistically significant.

## 3. Results

### 3.1. GPR30 Is Overexpressed in GC Tissues and Associated with Poor Prognosis of GC Patients

**Figure 2:**
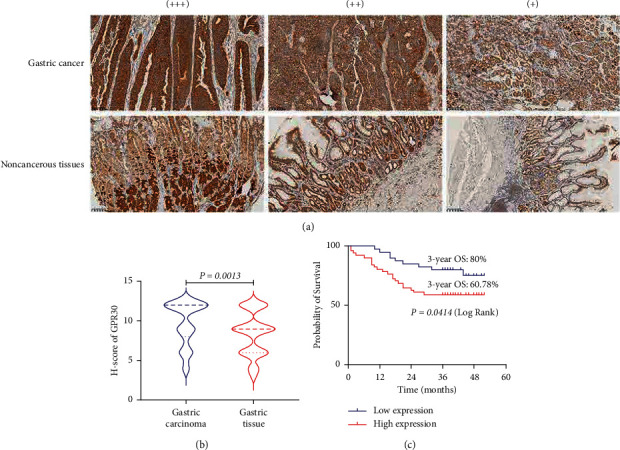
GPR30 is overexpressed in GC tissues compared with adjacent gastric tissues, and a high expression of GPR30 is associated with poor prognosis in GC patients. (a) Representative images of the GPR30 staining in TMAs as determined by immunohistochemical analysis (91 GC and 71 paired adjacent tissues). (b) Differential expression of GPR30 in GC and paired adjacent tissues in 91 GC patients. (c) The overall survival (OS) curves of GC patients with different GPR30 expression levels in GC, as determined by a Kaplan-Meier analysis (log-rank test).

To determine the expression of the GPR30 protein in GC tissues and their clinical significance, TMAs from 91 patients with GC were examined by immunohistochemical staining (Figure 2(a)). We found that 51 (56.04%) GC patients had a high expression of GPR30 in tumor tissues, and 40 (43.96%) patients had a low expression. However, there were 17 (23.94%) GC patients with a high GPR30 expression in adjacent normal tissues, and 54 (76.06%) patients with weak staining for GPR30 (all *P* < 0.001) (Table 1). Moreover, there was a significant difference between GC and paired adjacent tissues in GPR30 expression (Figure 2(b)), which suggested that high expression of GPR30 was associated with GC.

Next, we compared the clinicopathological features of GC patients with the expression level of GPR30 and found that GPR30 overexpression was significantly correlated with tumor size (Table 2). We also observed an excellent correlation between GPR30 expression and overall survival (OS). Patients with high GPR30 expression had a significantly poorer OS than those with low GPR30 expression. The 3-year OS rate of GC patients with low expression of GPR30 was 80%, while that of GC patients with high expression of GPR30 was only 60.78% (*P*=0.0414) (Figure 2(c)).

### 3.2. GPR30 Is Involved in the Migration and Invasion of GC Cells

**Figure 3:**
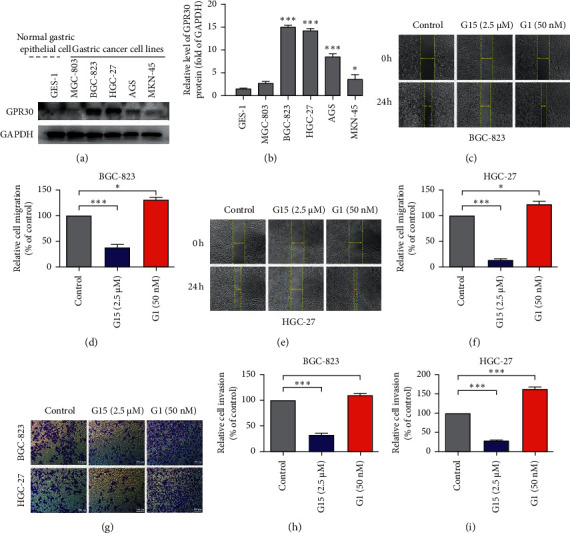
GPR30 is involved in the migration and invasion of GC. (a) Western blotting analysis of GPR30 expression in normal gastric epithelial cells and GC cells. The data in (b) was presented as the mean ± SEM. ^*∗*^*P* < 0.05, ^*∗∗∗*^*P* < 0.001, compared with GES-1. (c, e) A wound healing assay investigated the effects of G15 and G1 on the migration of BGC-823 (c) and HGC-27 cells (e). Representative photographs showed the same area at 0 h and 24 h after drug administration. (g) Transwell assay on the effects of G15 and G1 on the invasion of BGC-823 and HGC-27 cells. The representative photographs show 48 h after administration. (d–i) Wound healing (d, f) and transwell (h, i) assays were quantitatively analyzed by Image J software. ^*∗*^*P* < 0.05, ^*∗∗∗*^*P* < 0.001, compared with the control group.

Based on the results of IHC analysis, we investigated the expression of GPR30 in normal gastric epithelial cell line GES-1 and GC cell lines such as MGC-803, BGC-823, HGC-27, AGS, and MKN-45. We found that the expression of GPR30 in GC cells was higher than that in normal cells (Figures 3(a) and 3(b)). These data suggested GPR30 may have an important role in the development of GC.

Next, GPR30 selective antagonist G15 and agonist G1 were used to verify this assumption. Our previous studies had confirmed that 2.5 *μ*M G15 and 50 nM G1 can effectively block or activate GPR30 without obvious toxicity to cells [4]. After being treated with G15 for 48 h, the migratory (Figures 3(c)–3(f)) and invasive (Figures 3(g)–3(i)) ability decreased and increased after G1 treatment in BGC-823 and HGC-27 cells, indicating that GPR30 might regulate the biological behavior of GC cells.

### 3.3. NEH Inhibits Gastric Cancer In Vivo

**Figure 4:**
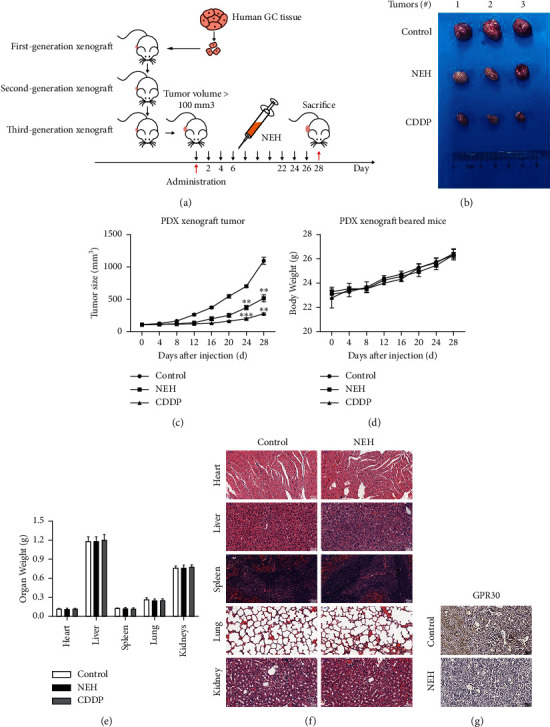
NEH inhibited gastric cancer in the PDX model. (a) Flowchart on the construction of PDX model and antitumor test of NEH *in vivo*. (b) Representative image of negative control, positive control (cisplatin/CDDP, 5 mg/kg), and NEH (25 mg/kg) treated tumors. *n* = 3 per group. (c) The volume of the tumors changed throughout the experiment. Volume = length × width^2^ × 1/2. Changes in body weight (d) and organ weight of major organs (e) in mice. (f) H&E staining of main organs of mice. (g) Immunohistochemical analysis of GPR30. ^*∗∗*^*P* < 0.01, ^*∗∗∗*^*P* < 0.001, compared with the control group.

We examined whether NEH has antitumor activity on GC *in vivo*. After establishment of the PDX mouse model, we investigate the anticancer activity of NEH in mice (Figure 4(a)). After NEH treatment, the tumor size decreased by about 52.92% (Figures 4(b) and 4(c)). The drug did not affect body weight (Figure 4(d)) and major organs such as heart, liver, and spleen of the mice (Figure 4(e)). Additionally, histological examination by H&E staining showed no morphological abnormalities and pathological changes in these organs (Figure 4(f)). In this experiment, cisplatin (CDDP) was used as a positive control drug. Cisplatin had a significant inhibitory effect on tumor growth. Interestingly, we also found that NEH significantly reduced the expression of GPR30 (Figure 4(g)). These results showed that NEH could effectively and specifically inhibit tumor growth without affecting other organs.

### 3.4. NEH Inhibits the Proliferation, Migration, and Invasion of GC Cells

**Figure 5:**
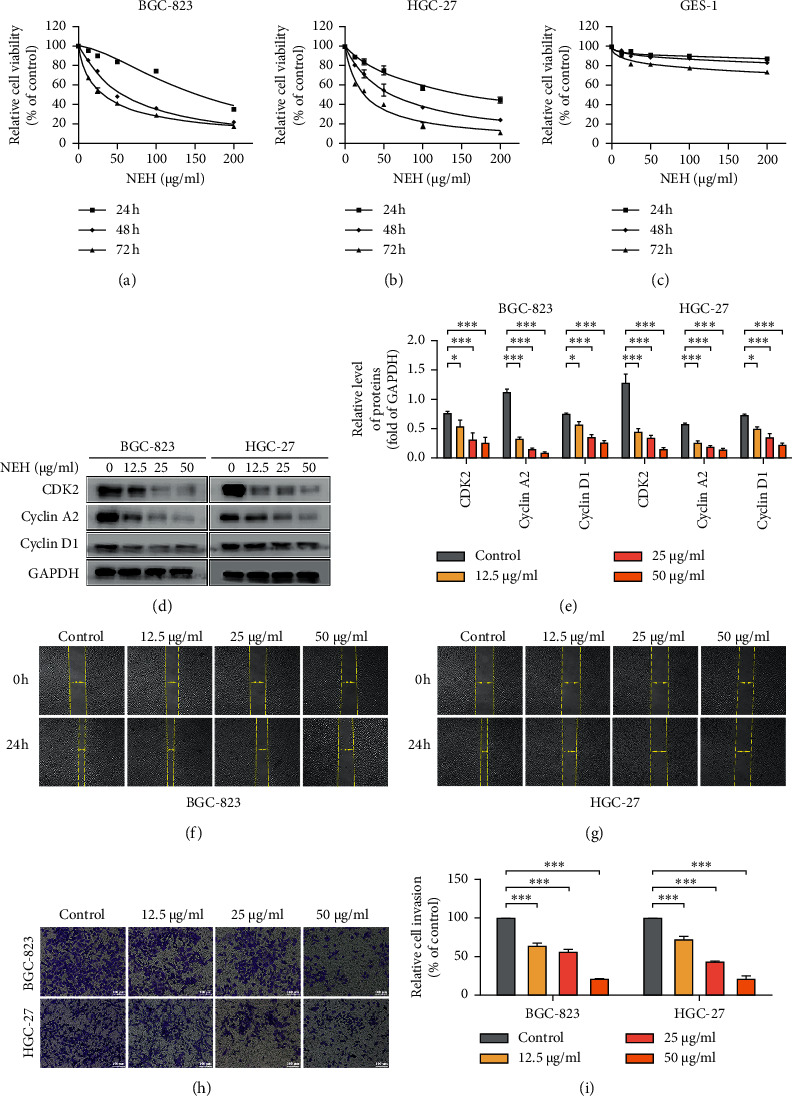
NEH inhibits the proliferation, migration, and invasion of GC cells. (a–c) CCK-8 assay performed to show the inhibitory effect of different concentrations (0, 12.5, 25, 50, 100, and 200 *μ*g/ml) of NEH on the proliferation of BGC-823 (a), HGC-27 (b), and GES-1 (c) cells. (d) Western blot analysis detected the main factors in the S phase and G2/M phase: CDK2, cyclin A2, and cyclin D1. The data in (e) was presented as the mean ± SEM. ^*∗*^*P* < 0.05, ^*∗∗∗*^*P* < 0.001, compared with the control group. (f, g) A wound healing assay investigated the effects of NEH on the migration of BGC-823 (f) and HGC-27 (g) cells. (h-i) Transwell assay on the effects of NEH on the invasion of BGC-823 and HGC-27 cells. Representative photographs show 48 h after drug administration. ^*∗∗∗*^*P* < 0.001, compared with the control group.

We further verified the inhibitory effect of NEH on GC cells *in vitro*. CCK-8 assay was used to determine the inhibitory effect of different concentrations (0∼200 *μ*g/ml) of NEH on the growth of normal gastric epithelial cells and GC cells at 24 h, 48 h, and 72 h. The results showed that NEH inhibited the growth of GC cells in a dose- and time-dependent manner. The IC_50_ value at 24, 48, and 72 h after treatment with NEH for BGC-823 cell was 153, 56.48, and 31.26 *μ*g/ml, and for HGC-27 cell, it was 153.1, 60.36, and 25.64 *μ*g/ml, respectively (Figures 5(a) and 5(b)). However, the survival rate of GES-1 cells was not affected (Figure 5(c)), suggesting that NEH affected GC cells and had low cytotoxicity on normal gastric epithelial cells.

Our previous studies indicated that NEH induced cell cycle arrest in the S phase and G2/M phase [15], so we detected the expression of its related proteins. The results showed that the expression of cell cycle-related proteins CDK2, cyclin A2, and cyclin D1 decreased after NEH treatment (Figures 5(d) and 5(e)). In addition, wound healing and transwell assay were used to determine the effect of NEH on the migration and invasion of GC cells. As shown in Figures 5(f)–5(i), the migration and invasion ability of GC cells were significantly inhibited compared with the untreated BGC-823 and HGC-27 cells after NEH treatment. These results showed that NEH inhibited the proliferation, migration, and invasion of GC cells.

### 3.5. The Inhibitory Effect of NEH on the Proliferation, Migration, and Invasion of GC Cells May Be Related to GPR30

**Figure 6:**
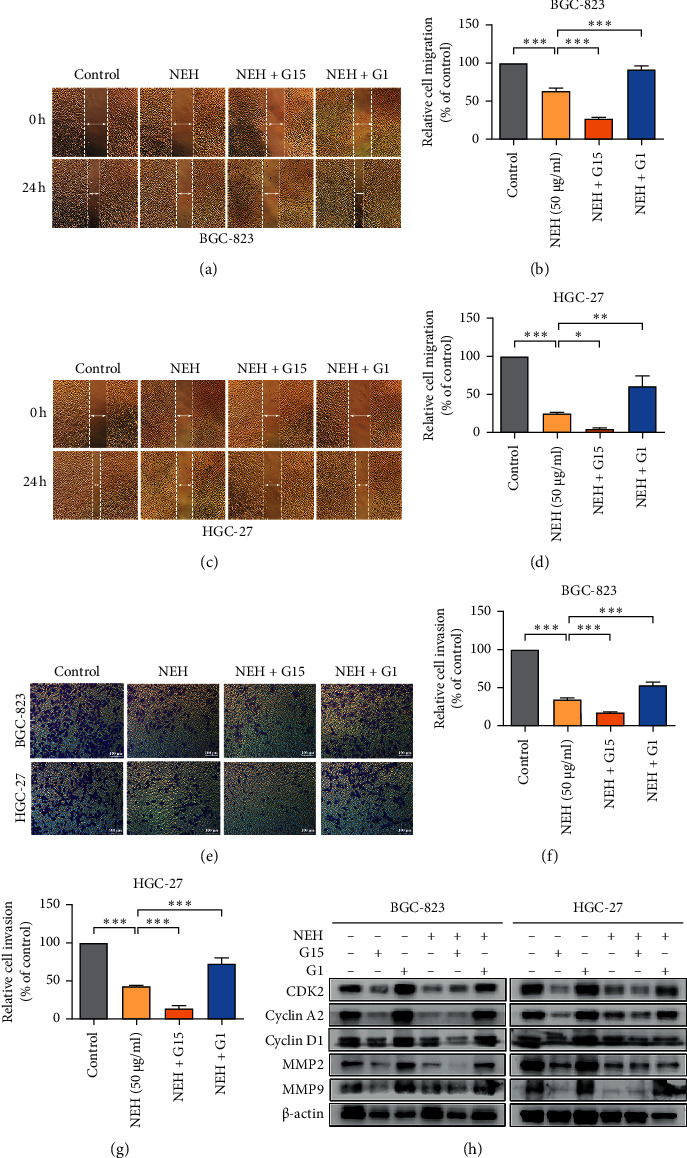
NEH inhibits the proliferation, migration, and invasion of GC cells through GPR30 signalling. (a–d) A Wound healing assay investigated the effects of NEH (50 *μ*g/ml), G15 (2.5 *μ*M), and G1 (50 nM) on the migration of BGC-823 (a, b) and HGC-27 (c, d) cells. (e–g) Transwell assay on the effects of NEH, G15, and G1 on the invasion of BGC-823 and HGC-27 cells. The representative photographs show 48 h after drug administration. ^*∗*^*P* < 0.05, ^*∗∗*^*P* < 0.01, and ^*∗∗∗*^*P* < 0.001, compared with the NEH-treated group. (h) Western blot analysis on the expression of CDK2, cyclin A2, cyclin D1, MMP2, and MMP9.

To investigate the role of GPR30 in the antitumor effect of NEH, low concentrations of G15 and G1 were used for further verification. As shown in Figures 6(a)–6(g), G15 further inhibited the migration and invasion of GC cells by the combination with NEH, while G1 eliminated this effect.

In order to further verify these results, a Western blot assay was used to detect the expression of MMP2 and MMP9. The results were consistent with the migration and invasion tests. In addition, our data showed that GPR30 was also involved in the effect of NEH on the cell cycle (Figure 6(h)). Overall, these results suggested that the effects of NEH on the proliferation, migration, and invasion of GC cells might be related to targeting GPR30.

### 3.6. NEH Inhibits the GPR30-Mediated PI3K/AKT Signalling Pathway

**Figure 7:**
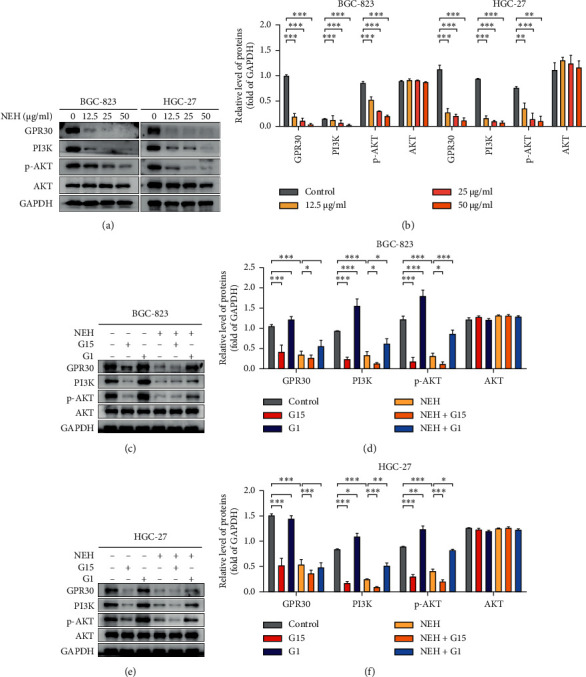
NEH inhibits the GPR30-mediated PI3K/AKT signalling pathway. (a) Western blot analysis on the influence of NEH on GPR30 and molecules on the PI3K/AKT signalling pathway. The data in (b) were presented as the mean ± SEM. ^*∗∗∗*^*P* < 0.001, compared with the control group. (c–f) Western blot analysis on the effects of NEH, G15, and G1 on the molecules of the GPR30/PI3K/AKT signalling pathway in GC cells. ^*∗*^*P* < 0.05, ^*∗∗*^*P* < 0.01, ^*∗∗∗*^*P* < 0.001, compared with the control group or NEH-treated group.

In order to clarify the specific mechanism of anti-GC effect of NEH, we investigated the effect of NEH on GPR30-mediated PI3K/AKT signalling pathway. As shown in Figures 7(a) and 7(b), after being treated with NEH for 48h, GPR30 and PI3K and the phosphorylation of AKT were inhibited in GC cells in a dose-dependent manner. G15 inhibits PI3K/AKT signalling pathway by blocking GPR30 signalling, while G1 activates PI3K/AKT signalling pathway. G15 combined with NEH further inhibited the PI3K/AKT signalling pathway, while G1 combined with NEH eliminated the inhibition of NEH on the PI3K/AKT signalling pathway. In addition, we also found that G15 also inhibited the expression of GPR30 (Figures 7(c)–7(f)). These results suggested that NEH may exert an anti-GC effect through the GPR30-mediated PI3K/AKT signalling pathway.

### 3.7. The Acute Toxicity of NEH in Mice

After a single intraperitoneal injection of a series of concentrations of NEH, some of the mice that received the highest concentration (800 mg/kg group) showed a temporary decrease in activity, which returned to normal after 30 min. There was no abnormality found in the other groups. The activity and weight gain of all mice were normal (Figures 1(b) and 1(c)), and no toxicity and death were observed. The organ weight of different organs showed similar normal values in all groups (Figures 1(d) and 1(e)).

In order to further clarify the biotoxicity of NEH, we used maximum intraperitoneal dose (0.4 ml/10g) and maximum dissolved concentration (40 mg/ml) of NEH into mice to test the maximum tolerance dose (MTD; 1600 mg/kg). The mice in the NEH group showed similar behavioral changes as the previous test; a few mice in the solvent control group showed slight mental malaise, and there was no significant change in the mice of the blank control group. No toxicity or death was found, and there was no obvious abnormality in the body weight of mice (Figures 1(f) and 1(g)).

The serum biochemical indexes of mice are shown in Table 3. The AST and Creatinine of male mice in the NEH group and solvent control group were higher than those in the blank control group. It was considered that DMSO might cause this. Besides, there was no significant difference in other serum biochemical indexes. In the pathological examination, no obvious pathological change was found (Figure 1(h)). These results suggested that NEH was not toxic, and the tolerance dose of NEH in mice was higher than 1600 mg/kg.

## 4. Discussion

GC is one of the most common types of cancers. Surgery is still the first choice for patients with early GC. However, most patients with GC have lost the choice of surgery because of distant metastasis. Therefore, comprehensive therapies are the main choices for advanced GC which include the chemotherapy, immunotherapy, targeted therapy, and TCMs. TCMs are widely used in healthcare in Asia, because of its unique advantages as anticancer.

GPR30 is located on the 7p22.3 chromosome and has biological function of regulation of endocrine, immune, neuronal, and cardiovascular functions. And it is also related to the occurrence and development of cancer, including the regulation of cell proliferation, cell apoptosis, tumor microenvironment, and metastasis [19]. Several studies showed that GPR30 activation could promote the proliferation of cancer cells via upregulating expression of cyclin D1, cyclin E, and cyclin A [20, 21]. In this study, we found that GPR30 was highly expressed in GC cells and tissues. And the higher expression level of GPR30 was correlated with poorer prognosis. And we also observed that GPR30 could regulate the expression of cyclin A, CDK2, and cyclin D1 in GC cells. In addition, some studies have confirmed that the expression level of GPR30 was closely related to the migration and metastasis [22]. And our previous studies confirmed that GPR30 was the upstream factor of EMT, and inhibition of GPR30 could reverse the EMT process of GC cells [4]. In the current study, we found that GPR30 expression could promote the migration and metastasis of GC cells and regulate the expression of MMP-2 and MMP-9. It reminded us that GPR30 activation may play a significant role in GC.

Recently, Huaier has been widely concerned due to its effective antitumor and immunomodulatory effects. Our previous study found that NEH with 51.4% total flavonoids can effectively inhibit the proliferation and metastasis of GC cells [15]. A large number of preclinical and clinical studies have shown that flavonoids have superior prevention and treatment in various cancers [23, 24]. In this study, we constructed a GC PDX mice model to confirm the effect of NEH *in vivo*. The result showed that NEH significantly inhibited the growth without obvious toxicity. In addition, we found that NEH could inhibit the proliferation, migration, and invasion ability of GC cells and downregulate the expression of CDK2, cyclin A2, cyclin D1, MMP2, and MMP9 in vitro. We further found that NEH inhibited the activation of PI3K/AKT signalling pathway by downregulating the expression of the PI3K and the phosphorylation of AKT. Besides, GPR30 selective antagonist G15 could promote the anti-GC effect of NEH, while GPR30 agonist G1 could eliminate this effect, indicating that the GPR30 is important for NEH's anticancer efficacy.

## 5. Conclusions

In conclusion, our research demonstrated that GPR30 plays a significant role in the progression of GC, and NEH inhibits the proliferation, migration, and invasion of GC cells through the PI3K/AKT signalling pathway mediated by GPR30. Thus, GPR30 may be a potential molecular target for GC, and NEH could be a new drug candidate for the treatment of GC.

## Figures and Tables

**Table 1:** Differential expression of GPR30 in GC and adjacent tissues.

Variables	*N*	GPR30 expression		Chi-square value	*P* value
		High	Low		
Tumor tissue	91	51	40	16.873	<0.001
Adjacent tissue	71	17	54		

^*∗*^Statistically significant (*P* < 0.05).

**Table 2:** The correlations between the GPR30 expression levels and the clinicopathological features of GC patients.

	GPR30 expression		Total	*χ*2	*P* value
	High	Low			
Age (year)					
<66	22	21	43	0.788	0.375
≥66	29	19	48		
Sex					
Female	14	5	19	3.033	0.082
Male	37	35	72		
Tumor size (cm)					
<5 cm	23	29	52	6.873	**0.009** ^*∗*^
≥5 cm	28	11	39		
Grade of differentiation					
Well/moderate	22	13	35	1.072	0.301
Poor/not	29	27	56		
T Stage					
T1/T2	4	7	11	1.163	0.281
T3/T4	47	33	80		
N stage					
N0/N1	18	17	35	0.492	0.483
N2/N3	33	23	56		
M Stage					
M0	48	40	88	2.433	0.119
M1	3	0	3		
TNM stage					
Ι/II	15	11	26	0.040	0.841
III/IV	36	29	65		
CEA (ng/ml)					
<5	41	32	73	0.002	0.963
≥5	10	8	18		
HER2					
Positive	7	2	9	1.061	0.303
Negative	44	38	82		
PD-1					
Positive	13	3	16	2.859	0.091
Negative	38	37	75		

^*∗*^ *P* < 0.05.

**Table 3:** Effects of NEH on blood biochemical indexes in mice.

Serum index	Group		
	Blank control	Solvent control	NEH
*Male*			
TP (g/L)	51.89 ± 1.42	52.67 ± 2.64	51.95 ± 1.61
ALB (g/L)	22.58 ± 1.03	23.40 ± 1.50	22.98 ± 0.70
ALT (U/L)	24.86 ± 4.16	28.92 ± 4.93	26.72 ± 6.18
AST (U/L)	83.76 ± 8.08	**112.56** ± **8.97**^*∗∗∗*^	**96.66** ± **15.86**^*∗*^
GLU (mmol/L)	9.64 ± 0.83	9.74 ± 0.79	9.52 ± 0.51
TG (mmol/L)	1.59 ± 0.21	1.86 ± 0.31	1.58 ± 0.29
CHO (mmol/L)	3.40 ± 0.56	3.53 ± 0.39	3.70 ± 0.22
BUN (mmol/L)	8.50 ± 1.28	8.49 ± 0.83	7.50 ± 0.72
Crea (*μ*mol/L)	10.84 ± 1.06	**12.50** ± **1.35**^*∗∗*^	**12.12** ± **1.14**^*∗*^

*Female*			
TP (g/L)	52.56 ± 1.42	53.11 ± 1.80	53.65 ± 2.63
ALB (g/L)	24.05 ± 0.73	24.38 ± 0.78	24.49 ± 1.20
ALT (U/L)	25.34 ± 3.40	24.86 ± 1.47	25.8 ± 7.34
AST (U/L)	114.56 ± 10.63	108.64 ± 9.09	114.72 ± 19.26
GLU (mmol/L)	8.17 ± 0.65	8.61 ± 0.96	8.44 ± 0.87
TG (mmol/L)	1.99 ± 0.24	2.59 ± 0.61	2.22 ± 0.27
CHO (mmol/L)	2.59 ± 0.27	2.59 ± 0.31	2.57 ± 0.35
BUN (mmol/L)	7.55 ± 1.27	7.46 ± 0.95	7.80 ± 0.45
Crea (*μ*mol/L)	11.92 ± 0.83	13.20 ± 1.61	12.34 ± 2.05

^*∗*^ *P* < 0.05, ^*∗∗*^*P* < 0.01, ^*∗∗∗*^*P* < 0.001.

## Data Availability

The datasets used and/or analyzed during the present study are available from the corresponding author upon reasonable request.
